# An application of Bayesian measurement invariance to modelling cognition over time in the English Longitudinal Study of Ageing

**DOI:** 10.1002/mpr.1749

**Published:** 2018-10-23

**Authors:** Benjamin David Williams, Tarani Chandola, Neil Pendleton

**Affiliations:** ^1^ Cathie Marsh Institute for Social Research University of Manchester Manchester UK; ^2^ Division of Neuroscience and Experimental Psychology University of Manchester Manchester UK

**Keywords:** approximate measurement invariance, cognitive function, ELSA, old age, statistics

## Abstract

**Objectives:**

Recommended cut‐off criteria for testing measurement invariance (MI) using the comparative fit index (CFI) vary between −0.002 and −0.01. We compared CFI results with those obtained using Bayesian approximate MI for cognitive function.

**Methods:**

We used cognitive function data from Waves 1–5 of the English Longitudinal Study of Ageing (ELSA; Wave 1 *n* = 11,951), a nationally representative sample of English adults aged ≥50. We tested for longitudinal invariance using CFI and approximate MI (prior for a difference between intercepts/loadings ~N(0,0.01)) in an attention factor (orientation to date, day, week, and month) and a memory factor (immediate and delayed recall, verbal fluency, and a prospective memory task).

**Results:**

Conventional CFI criteria found strong invariance for the attention factor (CFI + 0.002) but either weak or strong invariance for the memory factor (CFI −0.004). The approximate MI results also supported strong MI for attention but found 9/20 intercepts or thresholds were noninvariant for the memory factor. This supports weak rather than strong invariance.

**Conclusions:**

Within ELSA, the attention factor is suitable for longitudinal analysis but not the memory factor. More generally, in situations where the appropriate CFI criteria for invariance are unclear, Bayesian approximate MI could alternatively be used.

## 1 INTRODUCTION

Measurement invariance (MI) is an often‐underappreciated problem in psychiatric research. Although some outcomes in psychiatry are discrete and directly observable, many are impossible to observe directly. Cognitive function is an example of this and is the focus of this study. Latent variable analysis is one common method used to combine multiple measures into a single measure of an underlying concept of interest. However, a frequently occurring problem when using latent variables longitudinally is that the association between the observed variables and the unobserved latent variable changes over time.

In tests of cognition, performance on the tests will be determined the individual's ability in the target function (say working memory) but also their ability in a range of other cognitive and physical functions (such as attention and hearing). The demands on other functions will differ between tests. Each additional function utilised in performing each individual task may be differentially affected by ageing, disease, or setting (McAvinue et al., 2012; Wiegand et al., 2014). As well as different rates of change secondary to cognitive or physical processes, the size of practice effects may also vary between different tests of the same cognitive function (Calamia, Markon, & Tranel, 2012). Any of these may change the strength of the association between the individual cognitive tests and the latent cognitive function over time. In factor analysis, this manifests as a change in factor loading or intercept and is known as MI (van de Schoot, Lugtig, & Hox, 2012).

MI has been discussed extensively elsewhere and has been identified as a problem in longitudinal studies of cognitive function since at least the late 1980s and early 1990s (Horn & McArdle, 1992; Schaie, Willis, Jay, & Chipuer, 1989). With some notable exceptions, population and clinical research on cognitive function has had a tendency to overlook this issue with a preference for using summed scores, the measurement properties of which are often not examined (Blankson & McArdle, 2013; McArdle, Fisher, & Kadlec, 2007; Wicherts, 2016). If this issue is ignored, it biases estimates of change in cognitive function over time towards the direction of the change in latent intercept or varying effects for a change in factor loading (Ferrer, Balluerka, & Widaman, 2008; Horn & McArdle, 1992; van de Schoot et al., 2013; Wicherts, 2016; Widaman, Ferrer, & Conger, 2011). For example, practice effects would be expected to increase the intercept leading to an overestimation of cognitive ability at follow‐up visits and thus underestimation of decrease over time (Wicherts & Dolan, 2010). Alternatively, a decrease in factor loading due to increased sensory impairment over time weakening the association between measurable and latent cognitive function could lead to overestimation of cognitive function for low scorers and underestimation for high scorers as time progresses (Wicherts, 2016).

### 1.1 Conventional MI

Underlying a set of *k* (*n* = 0, …, *k*) continuous observed variables *c* that have been measured, there is a latent variable η (Muthén & Asparouhov, 2013; van de Schoot et al., 2013). If they are measured in individual *i* at time *t*, the measurement part is

(1) $$c_{\mathrm{ikt}}=v_{\mathrm{kt}}+\lambda_{\mathrm{kt}}\eta_{\mathrm{it}}+\varepsilon_{\mathrm{ikt}}.$$

Here, *c*_*ikt*_ is the observed value of variable *k* at time *t* in individual *i*, *v*_*kt*_ is the intercept for variable *k* at time *t*, *λ*_*kt*_ is the loading for variable *k* at time *t*, *η*_*it*_ is the value of the latent variable at time *t* for the variable *k*, and *ε*_*ikt*_ is the error for individual *i* at time *t* for observed variable *k*. This model assumes independence amongst the c's conditional on the factor that the residuals are uncorrelated with the factors and the errors are normally distributed with a mean of 0. The factor metric is usually set by fixing *λ* = 1 for one observed variable.

A linear growth curve for factor scores (the structural model) is

(2) $$\eta_{\mathrm{it}}=\eta_{\mathrm{oi}}+x_{t}\eta_{1t}+\varsigma_{\mathrm{it}}.$$

Here, *η*_0*i*_ is the intercept of the latent variable, *η*_1*i*_ is the slope growth factor, and *Ϛ*_*it*_ is the time and individual specific residual. The binary case is a straightforward extension of Equation (1), and if a probit link function is assumed, then the latent variable is assumed to follow a continuous distribution and the structural model is unchanged. Otherwise, it should be noted that the intercept *v*_*kt*_ is replaced with the threshold –*τ*_*tl*_ (Muthen, 2004).

For continuous variables, the specification of MI consists of (a) the same variables load onto the same factors at each time point (the same vector of *c*_*ikt*_ for each *η*_*it*_), (b) the factor loadings are equal at each time point (*λ*_*k*1_ = *λ*_*k*2_ = … = *λ*_*kt*_), (c) intercepts are equal at each time point (*v*_*k*1_ = *v*_*k*2_ = … = *v*_*kt*_), and (d) residual variances fixed across time (*ε*_*ik*1_ = *ε*_*ik*2_ = … = *ε*_*ikt*_; van de Schoot et al., 2012; Widaman et al., 2011). If only a holds, this is known as configural invariance, a–b weak invariance, a–c strong invariance, and a–d strict invariance. In the case of binary observed variables the second stage, weak factorial invariance is skipped because the item probability curve is influenced simultaneously by loading and intercept (Muthén & Muthén, 2014).

Strong invariance needs to be established in order to compare latent means over time (Ferrer et al., 2008; Widaman et al., 2011). If this assumption does not hold, then mean differences over time in a latent variable of cognitive function cannot be clearly attributed to change in true cognitive function because the scale of the dependent variable has changed. Additionally, tests of MI are sensitive to the choice of indicator variable (Shi, Song, Liao, Terry, & Snyder, 2017). This is fixed at 1 for every time point and is used to establish the scale of the latent variable, so at least one factor loading must be assumed to be invariant. Thus, the choice of a noninvariant reference variable can make decisions regarding MI significantly more difficult.

The standard approach to testing MI is to sequentially compare each level of increasing invariance using the χ^2^ test of model fit. With large sample sizes, this is a strict test and strong factorial invariance over time may be rejected even in robust longitudinal studies of cognitive ageing (Blankson & McArdle, 2013; Muthén & Asparouhov, 2013). Therefore, with large sample sizes, alternative fit indices, in particular the comparative fit index (CFI), are frequently used instead (Cheung & Rensvold, 2002; Meade & Bauer, 2007). However, recommendations for the change in CFI that establishes MI differ between studies, and these recommendations vary between 0.01 and 0.002 depending on author and the number of factors, indicators of those factors, and groups/occasions used (Chen, 2007; Cheung & Rensvold, 2002; Meade & Bauer, 2007; Meade, Johnson, & Braddy, 2008; Short, 2014).

Although these methods can be used to identify invariance, they are not informative about which parameters are invariant. To do this, one can either relax each equality constraint in turn or use modification indices, which give a measure of the improvement in model fit that would result from relaxing certain modelling assumptions. Relaxing each equality constraint sequentially means allowing each loading or intercept at each time point individually to be different to the same intercept or loading at all other time points. The change in model fit can then be assessed. This is laborious, and random variation can lead to different invariance solutions being identified depending upon the order in which the constraints are relaxed (MacCallum, Roznowski, & Necowitz, 1992; Muthén & Asparouhov, 2012). Modification indices are limited in application because they are only validated for two samples or time points (Muthén & Asparouhov, 2013).

### 1.2 Bayesian MI

Bayesian structural equation modelling (BSEM) has introduced the concept of approximate MI to take account of multiple small or moderate noninvariances in loadings, intercepts, or thresholds. Additionally, it provides a one‐step method of identifying which parameters are invariant (Muthén & Asparouhov, 2013; van de Schoot et al., 2013; Verhagen & Fox, 2013). The basic effect of approximate MI is that instead of requiring that all loadings be exactly equal, they are instead “tethered” so that they do not have to be exactly equal but are allowed to differ only by a substantively unimportant amount.

As described above, the conventional condition which must be met for strong factorial invariance (and therefore the ability to measure change in latent means over time) is that for each of the observed variables, *λ*_*k*1_ = *λ*_*k*2_ = … = *λ*_*kt*_ and *v*_*k*1_ = *v*_*k*2_ = … = *v*_*kt*_. Let ђ be the difference between λ's such that *λ*_*k*1_ − *λ*_*k*2_ = ђ_*k*12_, *λ*_*k*2_ − *λ*_*k*3_ = ђ_*k*23_, and *λ*_*k*1_ − *λ*_*k*3_ = ђ_*k*13_. Also, let be the difference between *v*'s such that ν_*k*1_ − ν_*k*2_ = и_*k*12_, ν_*k*2_ − ν_*k*3_ = и_*k*23_, and ν_*k*1_ − ν_*k*3_ = и_*k*13_. The conventional frequentist assumption of strong invariance can then be defined in Bayesian terms as the strongly informative priors of ђ_*kXX*_~*N*(0, 0) and и_*kXX*_~*N*(0, 0) (Muthén & Asparouhov, 2013).

Given that, from a Bayesian perspective, the factor loadings and intercepts are random variables, the assumption of 0 variance is difficult to envisage in this framework. With approximate MI, this is instead relaxed slightly to a still strong but more plausible informative prior with 0 mean and small variance such as ђ_*kXX*_~*N*(0,0.01) and и_*kXX*_~*N*(0,0.01). One reason for preferring the Bayesian approach in this situation is that this assumption of exact equality is relatively unrealistic in a number of situations due to issues such as random variation across many time points, attrition, or practice effects (Blankson & McArdle, 2013; Putnick & Bornstein, 2016). The researcher can decide a priori how long to make the tether by specifying an appropriate prior for the difference between loadings or intercepts over time. The size of the prior variance therefore sets the length of the tether and formalises the degree of invariance which is allowable.

The difference at each time point is tested to see whether it is statistically significantly different from the mean of the loadings at all time points. This tells you if any of the loadings have broken the tether and show a degree of noninvariance beyond that believed to be unimportant by the researcher. Additionally, this overcomes the problems in identifying the truly noninvariant parameters caused by fixing one indicator's loadings at 1 for all time points. Using the Bayesian approximate MI approach, one need only fix single loading for a single observed indicator at a single time point to 1 (Muthén & Asparouhov, 2013; Xu & Green, 2015).

An alternative frequentist approach to testing for MI is running models with and without MI to see if the results are conflicting (Widaman et al., 2011). With this approach, a, often informal, decision is made about the degree of conflict in the results that is acceptable before MI is rejected. This decision is made using substantive prior subject knowledge and implicitly includes an assumption about the acceptable degree of invariance. The Bayesian approach formalises the same substantive knowledge into the prior that can therefore be specifically tested.

When assessing for longitudinal invariance in the English Longitudinal Study of Ageing (ELSA), we encountered several of the aforementioned problems with conventional MI testing. The sample size is large, therefore the χ^2^ test likely to be overly conservative (Chen, 2007; Cheung & Rensvold, 2002; Steptoe, Breeze, Banks, & Nazroo, 2013). Additionally, as we will show, different cut‐offs for the CFI produced different conclusions. Moreover, the kind of invariance we were expecting was of multiple small deviations rather than few large deviations from invariance. Given the number of variables and time points in use, relaxing each constraint in turn would be both laborious and highly prone to the risk of error due to chance. For these reasons, we applied Bayesian approximate MI to test whether the conclusions about the level of MI drawn from this method differed to those drawn from the χ^2^ test and CFI rules.

Our primary research questions were first, in ELSA's cognitive function battery, is there longitudinal MI for an attention and a memory factor? Second, can Bayesian approximate MI be used to identify MI (or the lack thereof) in situations where CFI has an uncertain result?

## 2 METHODS

### 2.1 Participants and procedure

ELSA has been described in detail elsewhere (Steptoe et al., 2013). The study sample was drawn from participants in Health Survey for England years 1998, 1999, and 2001 who were born before March 1, 1952 and living in a private household or those in their households who were new partners or ≤50. We used the ELSA core sample that was nationally representative of the age specific English population at the time of recruitment. Data are collected in biennial sweeps by interview in the participants homes. For this analysis, data from Waves 1 (2002) to 5 (2010) were utilised because the core cognitive battery was consistent through this time.

Response rates at each wave were 70% at Wave 1, 82% at Wave 2, 73% at Wave 3, 74% at Wave 4, and 80% in Wave 5 (Steptoe et al., 2013). After the exclusion of extreme values (see below), final sample sizes at each wave were *n* = 11,951 in Wave 1, *n* = 9,313 in Wave 2, *n* = 7,850 in Wave 3, *n* = 6,911 in Wave 4, and *n* = 6,535 in Wave 5.

### 2.2 Cognitive measures

The cognitive tests were performed by computer‐assisted interview. Orientation to time was assessed by asking the participant to name the day, year, month, and date. To assess immediate and delayed veral recall, 10 common words were played to participants (Steel, Huppert, McWilliams, & Melzer, 2004). Immediate recall is assessed straight away, and delayed recall of the word list was tested after the other cognitive tests were undertaken (this also serve as a distraction technique). The word lists used were randomly assigned, and a standardised recording was used for all participants.

The prospective memory task required participants to remember to write their initials in the top corner of a page they were handed. Participants were prompted if they did not complete the actions spontaneously. A binary variable was used for remembering the correct action (either prompted or spontaneous). Semantic (category) fluency was assessed by asking participants to name as many animals as they can in 1 min. All the nonbinary variables were transformed to *z*‐scores for the purpose of inclusion in the factor structure.

### 2.3 Statistical analysis

Initially, extreme values with regard to the relationship between cognitive variables were identified by regressing each cognitive function variables in turn on all the others at each wave. The standardised residuals and leverage statistics were then compared and regression rerun with the exclusion of influential cases to see if the results were substantively different (Institute for Digital Research and Education, 2009). Only for month and year did the exclusion of high residual cases appears to make a substantive difference.

Due to the rarity of giving the incorrect response to year and month, almost all incorrect answers were considered extreme values by conventional recommendations. However, analysis of those cases with particularly high residual values identified a subset of cases who were incorrect on either year or month but achieved average or better results on all other tests. These cases were felt likely to represent either errors in recording or single item inattention. In total, 97 and 85 measurement occasions were excluded for year and month, respectively, meaning approximately 35 in total per wave of data collection. Other missing data were considered missing at random, which is as a property of the Bayesian estimation (Chen & Ibrahim, 2014). Research on how missingness affects longitudinal invariance has only been implemented in a single study using full information maximum likelihood and, while a topic warranting further investigation, is beyond the scope of this analysis (Sterba, 2017).

Initial exploratory factor analysis (EFA) and confirmatory factor analysis (CFA) assuming invariance were performed as part of an earlier study currently in submission. Two of the factors from this, attention (loaded onto by orientation questions) and memory (loaded onto by immediate and delayed recall, prospective memory, and verbal fluency), were used. The model was specified using CFA with configural invariance and modification indices checked to see if there was any need to make additional modifications beyond the basic factor structure (Muthén & Muthén, 2014). This identified that allowing residual covariance over time in verbal fluency and within factor covariances for immediate and delayed recall resulted in substantially improved model fit. This improved model was then tested using the χ^2^ test and CFI for MI.

Next, the Bayesian approximate MI model was specified. A prior variance of ~N(0,0.01) for all differences between loadings, intercepts, and thresholds at each wave with the mean across all waves was used. The MPlus default noninformative priors were used for all other model parameters (Muthén & Asparouhov, 2011). The conclusions about the level of MI in the data were then compared between frequentist χ^2^ test and CFI and Bayesian approximate MI.

The primary analysis was run for all ages in the ELSA data. Sensitivity analyses were run using age bands to check for one possible source of longitudinal noninvariance. Though there was slightly less noninvariance for older participants, and slightly more for younger participants, the overall pattern of results was very similar for all ages. Due to this, they are not presented here.

The data were edited using Stata version 12, and the structural equation modelling was performed using MPlus version 7.0 (Muthén & Muthén, 2014; StataCorp, 2011). Markov Chain Monte Carlo estimation was utilised with the MPlus default Gibbs sampler and convergence criterion, 105,000 iterations (of which the first 55,250 are burn‐in) and no thinning (Muthén & Asparouhov, 2011).

## 3 RESULTS

The participants at Wave 1 were 55.7% female, had a mean age of 64.2, and 2.8% of the sample were of non‐White ethnicity (Table 1). The large minority of participants were retired (47.7%), and the majority of the rest of the sample worked as either employed (28.1%) or self‐employed (5.7%). Most participants were married (56.2% first marriage; 11.1% remarried). The modal educational attainment was no qualifications (41.7%) with 11.5% having attained a degree. There was a bimodal distribution of social class with the largest groups being Class 5 (manual and routine occupations; 35.0%) and the second largest Class 1 (managerial and professional roles; 29.7%).

**Table 1:** Participant demographics at Wave 1

Variable		Total	Percentage
Age		64.2	*SD* 11.1
Female		6,676	55.7%
Non‐White		328	2.8%
Employment status			
Retired		5,715	47.7%
Employed		3,370	28.1%
Self‐employed		687	5.7%
Unemployed		123	1.0%
Permanent sick		783	6.5%
Homemaker		1,173	9.8%
Other		131	1.1%
Marital status			
1st Marriage		6,741	56.2%
Remarried		1,331	11.1%
Single		658	5.5%
Divorced/separated		1,256	10.5%
Widowed		2,003	16.7%
NS‐SEC social class			
1	Professional	3,487	29.7%
2		1,596	13.6%
3		1,223	10.4%
4		1,320	11.3%
5	Manual	4,112	35.0%
Highest qualification			
No qualifications		4,986	41.7%
High school		2,522	21.1%
6th Form		748	6.3%
Nondegree higher Ed.		1,317	11.0%
Degree		1,370	11.5%
Foreign qualification		1,014	8.5%

Cognitive function data were available for 11,630 of 11,951 participants at Wave 1; 9,066 of 9,313 at Wave 2; 7,659 of 7,850 at Wave 3; 6,656 of 6,911 at Wave 4; and 6,216 of 6,535 at Wave 5. The results showed a slight improvement in the memory factor tasks over time (Table 2). Mean immediate word recall was 5.4 (*SD* 1.8) in Wave 1 and 5.7 (*SD* 1.9) in Wave 5. Mean delayed recall was 4.0 (*SD* 1.8) in Wave 1 and 4.4 (*SD* 2.2) in Wave 5. The number of participants correctly remembering the prospective memory task was 79.3% in Wave 1 and 85.8% in Wave 5. The orientation to time tasks was stable over time. The proportion of participants in Wave 1 correctly identifying the year was 97.4%, date 80.6%, month 97.6%, and day 97.9%. This was not dissimilar to Wave 5 where the proportion of participants correctly identifying the year 97.3%, date 81.7%, month 97.8%, and day 97.5%.

**Table 2:** Mean or proportion of correct responses for each cognitive task

	Wave				
	1	2	3	4	5
*n*	11,630	9,066	7,659	6,656	6,535
	Mean[Link]				
Immediate	5.4 (1.8)	5.7 (1.8)	5.7 (1.8)	5.7 (1.8)	5.7 (1.9)
Delayed	4.0 (1.8)	4.3 (2.1)	4.4 (2.2)	4.4 (2.2)	4.4 (2.2)
Verbal fluency	19.3 (6.4)	19.8 (6.6)	19.8 (6.8)	20.2 (7.0)	20.2 (7.0)
	Proportion correct (%)				
Year	97.4	98.1	97.5	97.4	97.3
Date	80.6	81.4	80.8	80.8	81.7
Month	97.6	97.7	97.2	97.7	97.8
Day	97.9	97.8	97.6	97.7	97.5
Prospective	79.6	81.3	82.9	84.3	85.8

Results displayed as mean (standard deviation).

The factors structure was modelled based on previous EFA and CFA. The attention factor was composed of orientation to year, date, month, and day. The memory factor was composed of immediate and delayed recall, verbal fluency, and prospective memory. In the memory factor, the residual variances of verbal fluency were allowed to correlate over time and the residual variances between immediate and delayed recall were allowed to correlate at each time point, reflecting the more similar nature of these tasks.

When testing for longitudinal invariance using the χ^2^ test, all levels of MI were rejected for both the attention and memory factor with a *p* values of <0.001 (Table 3). We then compared the CFI between the models. The model with configural invariance both attention and memory had a CFI of 0.976. Setting strong invariance for the attention factor (not weak invariance as all four indicator variables were binary) actually improved the CFI to 0.978. Weak invariance for the memory factor also increased the CFI to 0.978 whereas strong invariance reduced it to 0.972. Strong invariance for both factors resulted in a CFI of 0.973 showing that the misfit induced by strong invariance in the memory factor was not compensated for by the improvement in fit from strong invariance in the attention factor.

**Table 3:** Model fit tests for conventional frequentist CFA

	χ^2^ test versus baseline model	χ^2^ test versus less restrictive model	CFI	CFI difference versus less restrictive model
All configural	<0.001	−	0.976	−
Attention strong	<0.001	0.002	0.978	0.002
Memory weak	<0.001	<0.001	0.978	0.002
Memory strong	<0.001	<0.001	0.972	−0.006
Both strong	<0.001	<0.001	0.973	0.001 or −0.005

*Note*. CFI: comparative fit index.

Therefore, using the CFI criteria, longitudinal MI was not rejected for the orientation factor by any criteria. On the other hand, the decrease in CFI of 0.006 in the change between weak and strong invariance for the memory factor falls between different recommendations from different studies.

The approximate MI results found that there was one parameter in the attention factor that showed a minor degree of noninvariance (Table 4); the 1st wave loading for recall of the day (0.326) that is 0.036 less than the mean loading across all waves (0.362); this was a statistically significant difference based on the 95% credible interval. This is not likely to have a substantively important impact on the results of longitudinal analysis.

**Table 4:** Factor loadings using Bayesian approximate measurement invariance for both factors at each time point

Item		Approximate invariance factor loadings (0.01 prior variance)					
		Wave 1	Wave 2	Wave 3	Wave 4	Wave 5	Mean
	Year	1	1.021	1.034	1.029	1.045	1.026
Orientation	Date	0.278	0.295	0.298	0.264	0.264	0.28
Factor	Month	0.51	0.54	0.555	0.513	0.516	0.527
	Day	**0.326** *	0.387	0.35	0.369	0.377	0.362
	Immediate recall	1	1.013	1.025	1.021	0.985	1.009
Memory	Delayed recall	1.08	1.102	1.101	1.082	1.064	1.086
Factor	Verbal fluency	0.856	0.897	0.896	**0.927** *	0.914	0.898
	Prospective mem.	0.88	0.934	0.875	0.911	0.855	0.891

		Approximate invariance intercepts^†^ and thresholds^‡^ (0.01 prior variance)					
		Wave 1	Wave 2	Wave 3	Wave 4	Wave 5	Mean
	Year^‡^	−5.887	−5.9	−5.892	−5.898	−5.89	−5.893
Orientation	Date^‡^	−1.095	−1.099	−1.088	−1.040	−1.062	−1.077
Factor	Month^‡^	−3.483	−3.446	−3.463	−3.457	−3.476	−3.465
	Day^‡^	−2.796	−2.853	−2.754	−2.847	−2.811	−2.812
	Immediate recall^†^	−0.013	**0.053** *	**0.033** *	−0.013	**−0.055** *	0.001
Memory	Delayed recall^†^	**−0.014** *	**0.069** *	**0.086** *	0.037	−0.013	0.033
Factor	Verbal fluency^†^	−0.011	**0.009** *	−0.013	−0.013	−0.063	−0.018
	Prospective Memory^‡^	**−0.963** *	−1.013	−1.034	−1.064	**−1.086** *	−1.032

* Statistically significant using 95% credible interval.

For the memory factor, there is only one noninvariant loading; the Wave 4 verbal fluency loading (0.927) that is 0.029 greater than the mean across all waves (0.898). However, 9 of the 20 intercepts and thresholds are noninvariant. For immediate recall, the 2nd (0.052 above the mean), 3rd (0.032 above the mean), and 5th (0.057 below the mean) loadings show significant noninvariance. For delayed recall, the 2nd (0.036 above the mean) and 3rd (0.053 above the mean) occasions are noninvariant. In verbal fluency, the 2nd measurement occasion is estimated as being 0.009 above the mean. For prospective memory task, the threshold on the 1st occasion is 0.069 above the mean and the 5th occasion is 0.054 below the mean for all measurement occasions.

This means that across multiple time points, there are different expected values of the indicator variables for memory when the mean of the factor is zero. Although the individual differences are small, the number of noninvariant parameters suggest that the latent mean at one time point is not directly comparable with another. It may be better not to use the memory factor for longitudinal analysis but to analyse the individual memory tasks separately. These results support the use of the stricter CFI criteria for MI in this case.

## 4 DISCUSSION

When analysing cognitive function data from ELSA, we encountered a situation where different recommendations for using the CFI to establish MI led to different conclusions. We sought to use approximate MI to provide an alternative method of deciding which level of MI to accept or reject. In this case, the approximate MI approach identified small but significant noninvariance in the loadings of the memory and attention factors that was not identified by the use of CFI (which did not reject weak invariance). However, the degree of invariance in loadings that was identified using approximate MI but missed by CFI was relatively trivial. This suggests that the assumptions of strong longitudinal MI in the attention factor and weak invariance in the memory factor are plausible.

The main source of longitudinal noninvariance was not in the factor loadings but the intercepts of the memory factor. This led to strong invariance to being rejected by both the stricter CFI criteria and approximate MI. This is particularly important because strong invariance is required to compare latent means over time and therefore necessary for longitudinal analysis. However, using alternative CFI cut‐off rules for MI would have led the authors to a different conclusion about the presence or absence of strong invariance for the memory factor. Using a cut‐off of −0.01 such as that recommended by Chen (2007) or Cheung and Rensvold (2002) would have suggested not rejecting strong MI. By the more stringent recommendations of Meade et al. (2008) of −0.002, strong but not weak invariance would have been rejected. Moreover, as discussed by Short (2014), the truly suitable cut‐off for CFI may be different again when using the specific number of time points and observed variables available. Using approximate MI revealed that there was a high proportion of noninvariant intercepts and thresholds for the memory factor caused by multiple small deviations from noninvariance. This would have been difficult to accurately identify in a step‐wise fashion using a frequentist estimator.

If using factor analysis or another data reduction method, including sum scores, then ignoring this MI would have resulted in bias in the estimation of the memory factor latent mean (Muthén & Asparouhov, 2013; van de Schoot et al., 2013). In our results, the Waves 2 and 3 memory factor latent means would have been overestimated due to increases in the immediate and delayed recall intercepts. Wave 5 would have been underestimated because of decreases in the immediate recall intercept and prospective memory threshold. These effects would result in bias in both the estimation of both the rate and shape of the latent growth curve.

The noninvariance in the memory factor seems to be a combination of several isolated deviations and a linked increase in immediate and delayed recall in Waves 2 and 3. It is possible that the noninvariance seen at Waves 2 and 3 for the intercepts of immediate and delayed recall represents unequal practice effects in the indicators of this factor. The reduction in Waves 4 and 5 may represent fatiguing practice effects, an initial practice effect followed by more rapid decline in performance on those tasks or practice effects for the other indicators catching up relative to the recall tasks (Calamia et al., 2012). Whether Bayesian MI could be used to detect non‐uniform practice effects may be an avenue for further research.

The present study has the strength of using data from a high‐quality multidisciplinary survey with a large sample size. This study is relevant to researchers with a wide variety of longitudinal research questions relating to phenomena that cannot be directly observed. It is especially pertinent for those researching common mental health disorders who wish to utilise the richness of multidisciplinary surveys but lack a validated measure (previously demonstrated to be invariance over time in the population of interest) of the construct of interest, as with cognition in the first five waves of ELSA. Here, difficulties due to a large number of small noninvariances are particularly likely to occur. Furthermore, the specific number of groups or time points may not to have been covered in previous simulation studies, thus the most appropriate cut‐off for the CFI or other fit indices not known.

The large sample size to some extent does cover one of the potential weaknesses of BSEM in that it can be highly sensitive to prior specifications (Depaoli, Yang, & Felt, 2017; van Erp, Mulder, & Oberski, 2018). Informative priors for one parameter have the property of inducing implicit priors for other covariant parameters in a fashion that is difficult to predict and manage (MacCallum, Edwards, & Cai, 2012). It should be noted that if there is insufficient data to generate informative priors, or they are not desired for substantive reasons, then BSEM estimates with noninformative priors tend to converge with maximum likelihood estimates (Helm, Castro‐Schilo, & Oravecz, 2017; Lee, Song, & Tang, 2007). Although the single step identification of noninvariant parameters offers significant theoretical advantages over methods such as modification indices, in terms of the reduction of the capitalization of chance in inferences, there are few simulation studies to confirm this finding (MacCallum et al., 2012).

BSEM retains the common practical problems of many types of Bayesians analysis in terms of computational intensity, challenges with assessing convergence, and unfamiliarity to many users. This is particularly the case in comparison with approaches to identifying MI such as straightforwardly comparing parameters between models that assume or do not assume MI. Although this approach may provide rapid answers in some clear‐cut situations, in many cases even if an acceptable difference between estimates is prespecified (e.g., 5% or 10%), the results are borderline (Flora & Curran, 2004). This approach will also be model specific if the target of interest is a predictor of growth or a distal outcome and the additional information about invariant parameters will not be obtained, unlike with approximate MI.

Approximate MI, although not a panacea, is designed to handle multiple small invariances, and its power to detect noninvariance is not known to be affected by changing the number of groups or occasions being compared, which provides substantial flexibility. As such, it may be useful for future researchers to consider when testing the measurement properties of their instruments in longitudinal research. With regard to ELSA specifically, we find an attention factor that essentially shows strong MI over time but only weak invariance for a memory factor. Although the degree of noninvariance was relatively small, it was on a large number of parameters and therefore, researchers may wish to either avoid using the memory factor for longitudinal research or accommodate the noninvariance using approximate or partial MI.
